# Characterization of sensory neuron subpopulations selectively expressing green fluorescent protein in phosphodiesterase 1C BAC transgenic mice

**DOI:** 10.1186/1744-8069-2-17

**Published:** 2006-05-08

**Authors:** Carole Torsney, Rebecca L Anderson, Kerry-Anne G Ryce-Paul, Amy B MacDermott

**Affiliations:** 1 Department of Physiology and Cellular Biophysics, Columbia University, NY, USA; 2 CT is currently in the Centre for Neuroscience Research, Division of Veterinary Biomedical Sciences, University of Edinburgh, UK; 3 RLA is currently in the Department of Anatomy & Histology and Centre for Neuroscience at Flinders University, Adelaide, Australia; 4 Center for Neurobiology and Behavior, Columbia University, NY, USA

## Abstract

**Background:**

The complex neuronal circuitry of the dorsal horn of the spinal cord is as yet poorly understood. However, defining the circuits underlying the transmission of information from primary afferents to higher levels is critical to our understanding of sensory processing. In this study, we have examined phosphodiesterase 1C (*Pde1c*) BAC transgenic mice in which a green fluorescent protein (GFP) reporter gene reflects *Pde1c *expression in sensory neuron subpopulations in the dorsal root ganglia and spinal cord.

**Results:**

Using double labeling immunofluorescence, we demonstrate GFP expression in specific subpopulations of primary sensory neurons and a distinct neuronal expression pattern within the spinal cord dorsal horn. In the dorsal root ganglia, their distribution is restricted to those subpopulations of primary sensory neurons that give rise to unmyelinated C fibers (neurofilament 200 negative). A small proportion of both non-peptidergic (IB4-binding) and peptidergic (CGRP immunoreactive) subclasses expressed GFP. However, GFP expression was more common in the non-peptidergic than the peptidergic subclass. GFP was also expressed in a subpopulation of the primary sensory neurons immunoreactive for the vanilloid receptor TRPV1 and the ATP-gated ion channel P2X_3_. In the spinal cord dorsal horn, GFP positive neurons were largely restricted to lamina I and to a lesser extent lamina II, but surprisingly did not coexpress markers for key neuronal populations present in the superficial dorsal horn.

**Conclusion:**

The expression of GFP in subclasses of nociceptors and also in dorsal horn regions densely innervated by nociceptors suggests that *Pde1c *marks a unique subpopulation of nociceptive sensory neurons.

## Background

Sensory information is conveyed from the periphery to the central nervous system via a heterogeneous population of primary sensory neurons that have their cell bodies in dorsal root ganglia (DRG). Sensory information is then processed within the complex neuronal circuitry of the dorsal horn before it is relayed to higher centers and 'perceived'. Key to our understanding of sensory processing is mapping the organization or 'wiring' of neurons within sensory pathways. This goal has recently been aided by the creation of mice expressing fluorescent markers within specific sensory neuron subpopulations [1-6]. Here we characterize mice in which green fluorescent protein (GFP) is specifically expressed in cyclic nucleotide phosphodiesterase 1C (*Pde1c*) positive cells using bacterial artificial chromosome (BAC) technology [7]. In the BAC transgenic vectors used to generate BAC transgenic lines, the endogenous messenger RNA and protein coding sequences of the gene of interest are replaced by sequences encoding a GFP reporter gene. As in any gene-replacement experiment, the stabilities of the reporter gene mRNA and protein can be different from those of the endogenous gene products. Thus, GFP expression reflects the relative rates of transcription of the gene of interest, in this case *Pde1c*, and is not a direct measure of mRNA or protein levels in BAC transgenic mice [7].

*Pde1c *is known to be involved in smooth muscle cell proliferation [8] but little is known about its function in the central nervous system. However, recent analysis of BAC transgenic mice has revealed that *Pde1c *marks populations of migrating neurons within the developing central nervous system, including the cerebellum and cerebral cortex [7]. Examination of the Gene Expression Nervous System Atlas (GENSAT) web database [9,10] further shows that *Pde1c *marks a subpopulation of spinal cord dorsal horn neurons. This, together with observed *Pde1c *protein levels in chick DRG [11], led us to characterize GFP expression in both the DRG and spinal cord dorsal horn of *Pde1C *BAC transgenic mice.

Here we report that GFP marks a subset of nociceptors in the DRG and also shows a distinct neuronal expression pattern within the superficial dorsal horn of *Pde1c *BAC transgenic mice.

## Results

### GFP-*Pde1c* expression in dorsal root ganglia

**Figure 1:**
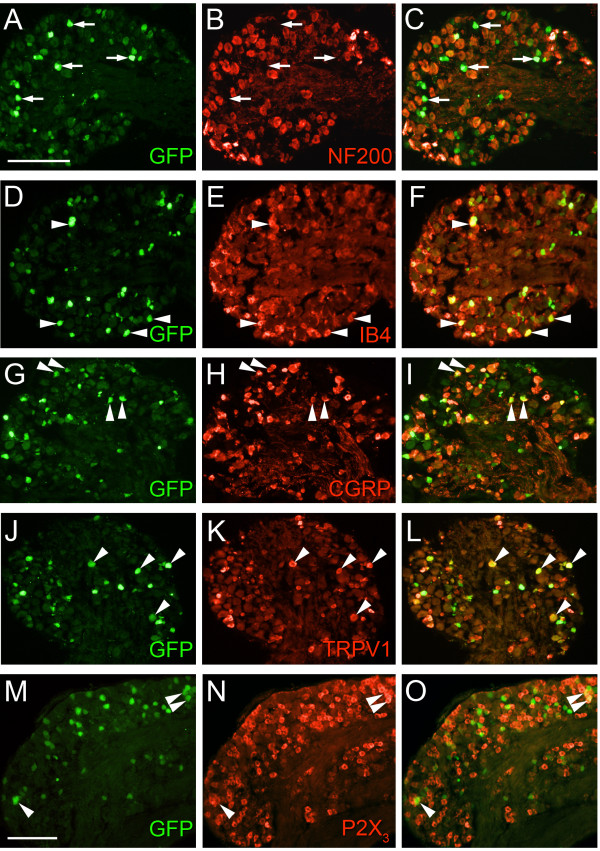
**GFP expression in peptidergic and non-peptidergic nociceptive primary sensory neurons in *Pde1c *BAC transgenic mice**. Lumbar DRG neurons from *Pde1c *BAC transgenic mice were stained with antibodies against GFP (green) to detect GFP-*Pde1c *expressing cells and with the lectin IB4 or antibodies against various sensory neuron markers (red). Arrows denote examples of GFP-immunoreactive cells negative for NF200 immunoreactivity (A-C). Arrowheads mark examples of GFP-immunoreactive cells double labeled for a given marker, evident in the overlay in the right panel (D-O). Images were taken using a wide-field fluorescence microscope. Scale bar, 100 μm in A applies to A-L; 100 μm in M applies to M-O.

**Figure 2:**
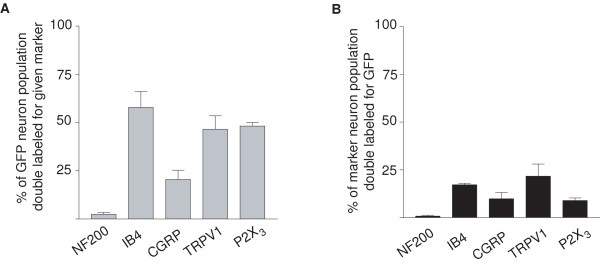
**Summary of GFP expression in DRG sensory neuron subpopulations in *Pde1c *BAC transgenic mice**. (A) The percentage of GFP-*Pde1c *expressing cells double labeled for a given marker. (B) The percentage of a given DRG sensory neuron subpopulation double labeled for GFP-*Pde1c*. Data are mean ± SEM (n = 3)

GFP-*Pde1c *expression was restricted to primary sensory neurons that give rise to unmyelinated C fibers. GFP-immunoreactive neuronal profiles in the DRG were rarely (<3%) immunoreactive for neurofilament-200 (NF200) a marker of primary sensory neurons that give rise to myelinated axons [12]. Conversely, NF200-immunoreactive DRG neurons were not (<1%) immunoreactive for GFP (Figure 1A–C and Figure 2). Unmyelinated C fibers, most of which are nociceptors, can be broadly subdivided into two classes 'peptidergic' and 'non-peptidergic' (reviewed in [13]). The peptidergic group expresses neuropeptides such as CGRP and requires NGF/trkA signaling for survival. In contrast, the non-peptidergic group lacks peptides, requires GDNF/c-RET signaling for survival and binds the plant lectin IB4. The non-peptidergic subclass express the ATP-gated ion channel P2X_3_whereas both groups of C fibers express the capsaicin TRPV1 receptor.

The majority of GFP-*Pde1c *expressing neurons belonged to the non-peptidergic subclass of nociceptors (~60% IB4-binding) with a smaller proportion belonging to the peptidergic subclass (~20% CGRP-immunoreactive; Figure 1D–I and Figure 2A). However, only a small subpopulation of either the IB4-binding (17%) or CGRP-immunoreactive (10%) DRG neurons were immunoreactive for GFP (Figure 2B). Interestingly, nearly 50% of GFP immunoreactive DRG neurons were immunoreactive for either the TRPV1 receptor or the P2X_3 _receptor (Figure 1J–O and Figure 2A) but again only a small proportion of TRPV1 receptor immunoreactive (22%) or P2X_3 _receptor immunoreactive (9%) neurons were immunoreactive for GFP (Figure 2B).

### GFP-*Pde1c* expression in spinal cord dorsal horn

**Figure 3:**
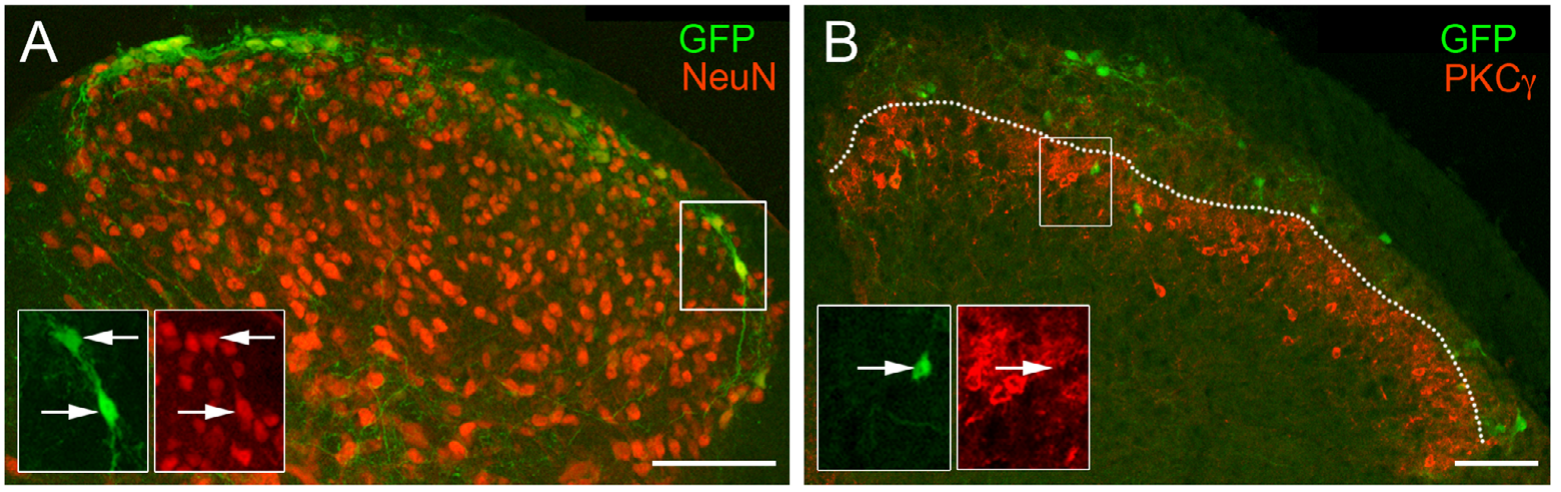
**Neuronal GFP expression in the superficial dorsal horn of *Pde1c *BAC transgenic mice**. (A) Overlay showing GFP-immunoreactivity (green) and NeuN-immunoreactivity (red) in spinal cord dorsal horn of a *Pde1c *BAC transgenic mouse. The insets (bottom left) show higher magnification images of the boxed area shown at right. Arrows show examples of double labeled profiles. (B) Merged images of GFP-immunoreactivity (green) and PKCγ-immunoreactivity (red) in the spinal cord dorsal horn. The insets (bottom left) show higher magnification images of the boxed area. Dotted line illustrates the displacement of the dense band of PKCγ-immunoreactivity from the dorsal aspect in the central portion. (A) and (B) are both montages of low magnification single confocal optical sections. Scale bar, 100 μm in each panel. Dorsal uppermost, medial left both panels.

**Figure 4:**
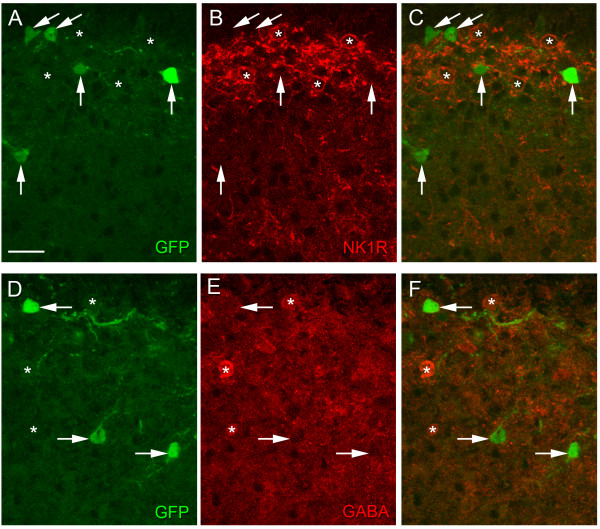
**GFP-*Pde1c *neurons in the superficial dorsal horn are not immunoreactive for NK1R or GABA**. High magnification single confocal optical sections of lamina I and II to show immunoreactivity to GFP (A, D) and either NK1R (B) or GABA (E). Overlays are shown in (C) and (F). Arrows denote GFP-immunoreactive neurons that are not immunoreactive for the respective marker. Asterisks show NK1R- or GABA-immunoreactive neurons that are not immunoreactive for GFP. NK1R-immunoreactive neurons appear as red circular rim staining because immunostaining is mainly associated with the cell membrane. Scale bar in A, 10 μm applies to all panels.

**Figure 5:**
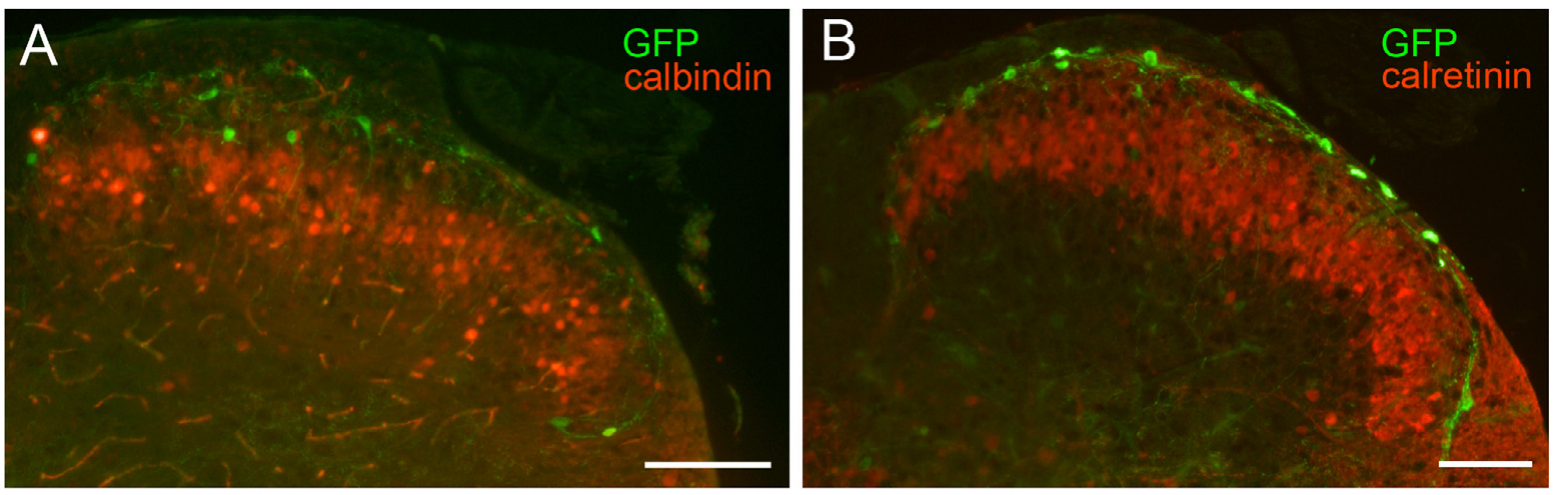
**GFP-*Pde1c *neurons in the superficial dorsal horn are not immunoreactive for calbindin or calretinin**. (A) and (B) show merged images of GFP-immunoreactivity (green) and calbindin-, or calretinin-immunoreactivity (red). (A) and (B) are overlays of single images taken using a wide-field fluorescence microscope. Scale bar, 100 μm in each panel. Dorsal uppermost, medial left both panels.

GFP-immunoreactive neurons were observed in the superficial dorsal horn of *Pde1c *BAC transgenic mice. GFP-immunoreactive profiles were demonstrated to be neuronal given that most (>85%) were immunoreactive for NeuN, a reliable marker of all spinal cord neurons [14] (Figure 3A). Given that NeuN is a neuronal nuclear protein, it seems more likely that the small numbers of profiles lacking NeuN-immunoreactivity reflect profiles not sectioned through the nucleus, rather than non-neuronal identity. We observed approximately 10 GFP-immunoreactive neurons, per dorsal horn, in each 20 μm section. Immunoreactive fibers were also observed (Figures 3, 4, 5) which may reflect axonal or dendritic processes of GFP-immunoreactive dorsal horn neurons or primary afferent terminals of the GFP-*Pde1c *nociceptors characterized above. Double labeling with NeuN reveals that GFP-*Pde1c *is predominantly expressed in the most superficial neurons in lamina I (Figure 3A). However, double labeling with PKCγ, which results in a dense plexus of immunostaining occupying the ventral part of lamina II [15] demonstrates that some GFP-*Pde1c *neurons also exist within lamina II (Figure 3B). Of interest, the central portion of the dense band of PKCγ-immunoreactivity is displaced from the dorsal aspect, likely reflecting the central thickening of lamina I previously reported in rat [14].

In an attempt to establish the identity of GFP-*Pde1c *spinal cord neurons, we performed double labeling immunofluorescence for GFP and well established neurochemical markers of key superficial dorsal horn neuron subpopulations, with a particular focus on lamina I. The neurokinin 1 receptor (NK1R) has been shown to be expressed by 45% of lamina I neurons but only 6% of neurons in lamina II [14] and therefore seemed a likely candidate. NK1R-expressing dorsal horn neurons are an excitatory population, since the vast majority, including all of those in lamina I, are not GABA-immunoreactive [16]. At high magnification, in single confocal optical sections, NK1R-immunoreactivity is observed as circular rim staining (Figure 4B) since the immunostaining is mainly associated with the cell membrane. To determine whether GFP-*Pde1c *neurons express the NK1R, optical sections were gathered in 1 μm z-steps through GFP-immunoreactive neurons and examined for NK1R-immunoreactivity. None of the GFP-immunoreactive neurons examined (~30 per animal) showed any evidence of immunoreactivity for NK1R, although NK1R-immunreactive profiles were clearly observed in the same optical sections (Figure 4A–C).

In a similar manner, GFP-immunoreactive neurons were examined for immunoreactivity to GABA. 28% and 31% of neurons in lamina I and II, respectively, have been shown to be immunoreactive for GABA [17]. To avoid confounding effects of autofluorescence when using antibodies raised against glutaraldehyde conjugates of GABA we used an antibody raised against a formaldehyde conjugate of GABA [see [18]]. This antibody has been shown to give a similar distribution of immunostaining as that seen with conventional antibodies raised against glutaraldehyde conjugates of GABA in rat spinal cord [14]. None of the GFP-immunoreactive neurons examined (~23 per animal) were immunoreactive for GABA, however GABA-immunoreactive profiles were evident in the same optical sections (Figure 4D–F). We were careful to examine GFP-immunoreactive neurons only in optical sections with clear GABA-immunostaining but given the potential penetration problems with GABA antibodies we cannot exclude the possibility of false negatives. However, the complete lack of overlap would tend to suggest that GFP-*Pde1c *expressing neurons are not inhibitory.

We then focused on markers of excitatory dorsal horn neurons other than the NK1R described above. PKCγ, somatostatin, neurotensin and MOR-1 have all been used as markers of excitatory dorsal horn neurons as they are not immunoreactive for GABA [15,19,20]. However, given that only PKCγ shows immunolabeling of neurons in lamina I, we focused on this particular marker. Neurons in lamina I and dorsal lamina II are weakly immunoreactive for PKCγ, compared with strong immunoreactive neurons in ventral lamina II/lamina III. In addition those in lamina I show some overlap with the above-mentioned NK1R-immunoreactive neurons [15]. Figure 3B shows the typical dense plexus of PKCγ immunostaining in the ventral part of lamina II. Analysis of GFP-immunoreactive neurons (~60 per animal) revealed that GFP-immunoreactive neurons in both lamina I and II were not immunoreactive for PKCγ (<1%). The calcium-binding proteins calbindin and calretinin have been reported to be present in superficial dorsal horn neurons in the rat [21]. In culture, it has been shown that dorsal horn neurons immunoreactive for calbindin and calretinin lack immunoreactivity for GABA suggesting that they may represent an excitatory neuron population [22]. We found that the overall distribution of calcium-binding protein immunolabeling in mice was somewhat different from that reported in rat. In rat, many lamina I and II neurons are calbindin-immunoreactive but in mice we found that while many lamina II neurons are calbindin-immunoreactive very few of those in lamina I express this calcium-binding protein (Figure 5A). The converse is true with respect to calretinin. In rats, many lamina II neurons but few lamina I neurons are calretinin-immunoreactive while in mice many calretinin-immunoreactive neurons were present throughout lamina I and II (Figure 5B). Irrespective of this overall difference in the expression pattern of calcium-binding proteins, close examination of GFP-immunoreactive neurons, (~60 per animal) for either marker demonstrated that GFP only rarely colocalized with calbindin (<4%) or calretinin (<3%).

## Discussion

We have demonstrated that in *Pde1c *BAC transgenic mice, GFP marks a subpopulation of nociceptors in the DRG. Expression of GFP-*Pde1c *was found to be more common in the IB4-binding, non-peptidergic subclass than the peptidergic subclass. The IB4-binding subclass of nociceptors has recently been the focus of much attention. Using genetically encoded tracers to mark *Mrgprd (Mas-related G protein-coupled receptor d)-*expressing neurons it was demonstrated that a major subpopulation of non-peptidergic neurons project exclusively to the skin and terminate in distinct epidermal regions [5]. More recently, using a tract tracing method in transgenic mice, it has been shown that non-peptidergic nociceptors are at the origin of a multisynaptic ascending pathway that targets limbic/affective brain regions [6]. Clearly, analysis of transgenic mice selectively expressing fluorescent markers in sensory neurons subpopulations or pathways has the potential to greatly expand our knowledge of somatosensory wiring.

*Pde1c *has also been observed in sensory neurons in the olfactory system [23]. Many odorants activate olfactory sensory neurons through G protein-coupled receptors that elicit a rapid and transient rise in cAMP levels [24]. *Pde1c *is postulated to play a role in the rapid termination of the odorant-induced cAMP signal, that is thought to be important for effective olfaction [23]. Nociceptive primary sensory neurons express an array of G protein-coupled receptors [25]. *Pde1c *may therefore have a similar role to play in somatosensory transduction and signaling.

We have also demonstrated that GFP marks a subpopulation of lamina I, and occasional lamina II neurons, in the spinal cord of *Pde1c *BAC transgenic mice. GFP-immunoreactive neurons were not, or only very rarely, double labeled for NK1R, GABA, PKCγ, calbindin or calretinin, markers of key neuronal subpopulations in the superficial dorsal horn. They were, however, double labeled with the neuronal nuclear protein NeuN, confirming a neuronal identity.

Recent studies have utilized the ability to fluorescently pre-identify and record from specific neuronal subpopulations in the live spinal cord slice preparation. The morphological, neurochemical and electrophysiological characteristics of a subpopulation of inhibitory superficial dorsal horn neurons, have been studied in transgenic mice expressing EGFP under the control of the GAD67 promoter [2,3]. A discrete subpopulation of inhibitory neurons, localized in ventral lamina II has also been extensively characterized in mice expressing GFP under the control of a mouse prion promoter [1,4]. In contrast, notwithstanding the possibility of false negatives, it would appear that GFP-*Pde1c *neurons represent a pre-identifiable population of excitatory neurons in the superficial dorsal horn.

Non-transgenic approaches have also been employed to fluorescently pre-identify dorsal horn neurons for electrophysiological recording. Lamina I projection neurons have been retrogradely labeled from specific brainstem regions [26-28] and superficial NK1R-expressing neurons have been identified using fluorescently-conjugated substance P [29-31]. Approximately 5% of lamina I neurons are projection neurons [32] and the vast majority (80%) of these are immunoreactive for the NK1 receptor [33,34]. Therefore, given that the GFP-*Pde1c *lamina I neurons were not NK1R-immunoreactive, it is unlikely that they are projection neurons. Moreover, we observed ~10 GFP-immunoreactive neurons, per dorsal horn, in each 20 μm section. This frequency greatly exceeds the 5% of lamina I neurons (1.6 cells/10 μm) that have been shown to project to higher centers [32].

GFP-*Pde1c *neurons may therefore represent a novel subgroup of dorsal horn neurons that can be fluorescently pre-identified in the mouse spinal cord. Their restricted expression in the superficial dorsal horn, which is densely innervated by nociceptors and predominant expression in lamina I neurons, most of which are nociceptive [35] suggests that they are likely to be involved in pain signaling in the dorsal horn.

The *Pde1c *BAC transgenic mice that were analyzed in the present study were a preliminary line that employed a GFP rather than an enhanced GFP (EGFP) reporter gene. It was therefore necessary to use antisera to GFP to amplify and visualize the reporter gene signal. However, at present, all mice in the GENSAT BAC transgenics project now employ an EGFP reporter gene and consequently the fluorescence signal can be viewed directly without amplification [7,9]. Notably, this should allow direct targeting of EGFP-*Pde1c *neurons for electrophysiological recording, in live tissue preparations, similar to other studies of EGFP expressing transgenic mice generated using BAC [36] and other approaches [1-4]. The present study focused on lumbar spinal cord and DRG levels because this axial level is the most extensively characterized with regards to neurochemical markers and sensory processing. It should be mentioned, however, that the GENSAT database [9] reveals that GFP-*Pde1c *neurons are also found in superficial dorsal horn layers at thoracic and cervical levels. Moreover, the database also shows that the dorsal horn expression pattern observed in P21 pups, in the present study, is broadly similar in P7 and adult animals. Importantly, it should be noted that as the reporter gene reflects the relative rates of transcription of the gene of interest and is not a direct measure of mRNA or protein levels this approach can reveal differences or novel expression not observed with in situ and immunohistochemical approaches [7,9].

## Conclusion

The BAC transgenic approach allows reproducible experimental access to specific neuronal subpopulations not previously available. In this particular BAC transgenic mouse line GFP-*Pde1c *shows a restricted expression pattern in nociceptive primary sensory neurons and dorsal horn neuronal regions which are targeted by nociceptors. *Pde1c*-BAC transgenic mice can therefore be utilised to enable direct access to 'nociceptive' pathways and further our understanding of pain processing.

## Methods

All procedures were in accordance with Columbia University Institutional Animal Care and Use Committee. Postnatal day (P) 21 *Pde1c *BAC transgenic mice were generously provided by Nathaniel Heintz and Mary E. Hatten of Rockefeller University (GENSAT BAC transgenic project). In these particular BAC transgenic mice a GFP reporter gene rather than enhanced green fluorescent protein (EGFP) was employed, necessitating the use of antisera to GFP to amplify the reporter gene signal in fixed tissue.

### Immunocytochemistry

Lumbar dorsal root ganglia (DRG) (L1-6) were obtained from P21 *Pde1c *BAC transgenic mice (n = 3) which had been deeply anesthetized with isoflurane, decapitated and dissected in cold phosphate buffered saline (PBS). Tissue was immersion fixed in 2% formaldehyde and 15% filtered saturated picric acid in 0.1 M phosphate buffer (PB), pH 7.3 at 4°C overnight, dehydrated (in 80% and 100% EtOH) and permeabilized (in DMSO) then cryoprotected in 30% sucrose in 0.1 M PB prior to cryostat sectioning. For comparative purposes, consecutive 10 μm sections were collected on sequential slides, allowing different antibody combinations to be tested on the same DRG and ensuring that sections on any given slide were at least 100 μm apart. Sections were blocked (1 h) in 10% normal goat/normal donkey serum in PBS. Antibody diluent contained 1% normal goat/normal donkey serum in PBS. Primary antibody incubation was overnight at room temperature and those in fluorescent secondary antibodies were 3 h. Double immunofluoresence labeling was performed with rabbit antiserum to GFP (1:1000 Molecular Probes, Eugene, OR) and biotinylated IB4 (10 μg/ml, Sigma, St.Louis, MO), mouse antiserum to NF200 (1:10,000, Sigma) or guinea-pig antiserum to P2X_3 _receptor (1:20,000, Neuromics, Northfield, MN). Sections were subsequently incubated in a mixture of Alexa 488 goat anti-rabbit IgG (1:500 Molecular Probes) and Streptavidin 568 (1:1000, Molecular Probes), Cy3 goat anti-mouse or guinea pig IgG (1:500, Jackson Immunoresearch, West Grove, PA). Double labeling was also performed with sheep antiserum to GFP (1:500, Biogenesis, Kingston, NH) and rabbit antisera to CGRP (1:12,000, Chemicon International, Temecula, CA) or TRPV1 (1:5,000 gift from D. Julius, University of California at San Francisco, CA) followed by FITC donkey anti-sheep (1:200, Jackson Immunoresearch) and Cy3 donkey anti-rabbit (1:800 Jackson Immunoresearch).

Lumbar spinal cord segments (L4-6) were obtained from P21 *Pde1c *BAC transgenic mice (n = 2) which had been deeply anesthetized with isoflurane then perfused, following a brief rinse, with 4% formaldehyde in 0.1 M PB (both at 37°C). Tissue was postfixed overnight then cryoprotected in 30% sucrose in 0.1 M PB prior to cryostat sectioning. Transverse sections (20 μm) were collected serially, 300 μm apart. Sections were blocked (1 h) in 10% normal goat/normal donkey serum in PBS with 0.1% Triton X-100. Antibody diluent contained 1% normal goat/normal donkey serum in PBS with 0.1% Triton X-100. Primary antibody incubation was overnight at room temperature (except for antisera to GABA, 48 hr at 4°C) and those in fluorescent secondary antibodies were 3 h. Double immunofluoresence labeling was performed with rabbit antiserum to GFP (1:1000 Molecular Probes) and mouse antisera to NeuN (1:1000, Chemicon) or calbindin (1:1000, Swant, Bellizona, Switzerland). Sections were subsequently incubated in a mixture of Alexa 488 goat anti-rabbit IgG (1:500 Molecular Probes) and Cy3 goat anti-mouse IgG (1:500, Jackson Immunoresearch). Double labeling was also performed with sheep antiserum to GFP (1:250, Biogenesis) and rabbit antisera to NK1 receptor (C terminus, 1:7500 gift from S. R. Vigna, Duke University Medical Center, Durham, NC), PKCγ (1:1000, Santa Cruz Biotechnology, Santa Cruz, CA) calretinin (1:5000, Swant) and paraformaldehyde conjugate of GABA (1:5000, gift from D. V. Pow, University of Queensland, Brisbane, Australia) followed by FITC donkey anti-sheep (1:200, Jackson Immunoresearch) and Cy3 donkey anti-rabbit (1:800 Jackson Immunoresearch).

### Analysis

Images of DRG sections were captured on a Nikon Eclipse E800 fluorescence microscope (×10 magnification) using a Nikon FDX-35 camera. GFP-*Pde1c *colocalization with each neuronal marker was performed by computer analysis using the MetaMorph Imaging System (Molecular Devices, Sunnyvale, CA). GFP immunoreactive neuronal profiles (>10 μm) were identified and overlayed images (FITC and Cy3, with each marker analyzed) were used to count double labeled neurons. To determine the proportion of a given marker population expressing GFP, positively immunostained neuronal profiles (neurons with a clearly identifiable nucleus) for each marker were identified and the overlayed images were used to count double labeled neurons. Three independent observers counted the number of double-labeled neurons in 5/6 sections per animal for each marker. In each animal an average of ~70 GFP immunoreactive neuronal profiles were examined for each marker and for each marker an average of ~130 cells were assessed for co-labeling with GFP. Counts were averaged across observers to obtain a single percentage of GFP double labeled neurons for each marker for each individual animal. Three separate averages (*n *= 3 animals) were expressed as the mean ± SEM to give the final values.

For spinal cord analysis, colocalization of GFP-*Pde1c *with NeuN, PKCγ calbindin or calretinin was assessed using a Nikon Eclipse E800 fluorescence microscope (×40 magnification) connected to a Nikon FDX-35 camera. Approximately 60 GFP-*Pde1c *positive cells, across 3 sections per animal were assessed for each marker. Colocalization of GFP-*Pde1c *with NK1 receptor or GABA was examined using a confocal laser scanning microscope (LSM 510 Meta, Carl Zeiss Inc.). Series of images through the mediolateral extent of the superficial dorsal horn were gathered in 1 μm z-steps using a × 40 objective lens. Individual optical sections through GFP-*Pde1c *cells were examined to determine whether they were also immunoreactive for NK1 receptor (~30 GFP cells per animal) or GABA (~23 GFP cells per animal).

Adobe Photoshop CS2 (Adobe Systems, Mountain View, CA) was used to prepare figures. Images were false-colored and the brightness and contrast adjusted.

## Competing interests

The author(s) declare that they have no competing interests.
